# The Efficacy of Psychosocial Interventions in Minimising the Harm Caused to Affected Others of Problem Gambling: A Systematic Review and Meta-Analysis

**DOI:** 10.1007/s10899-023-10220-3

**Published:** 2023-06-09

**Authors:** Matthew Vassallo, Katya DeGiovanni, Paul Montgomery

**Affiliations:** 1 Faculty for Social Wellbeing, University of Malta, Msida, Malta; 2 School of Social Policy, University of Birmingham, Birmingham, UK

**Keywords:** Systematic review, Problem gambling, Concerned significant others, Treatment, Intervention, Synthesis

## Abstract

Problem gambling not only impacts gamblers but also protrudes onto several affected others who experience adverse impacts, including financial, health, relationships, and psychological problems. The aims of this systematic review were twofold; to identify the psychosocial interventions to minimise the harm caused to affected others of problem gambling and to assess their efficacy. This study was conducted as outlined in the research protocol PROSPERO (CRD42021239138). Database searches were conducted in CENTRAL, MEDLINE, Social Science Database, CINHAL Complete, Academic Search Ultimate and PsycINFO. Randomised controlled trials of psychosocial interventions that aimed to minimise the harm caused to affected others of problem gambling written in English were eligible for inclusion. Risk of bias for included studies was assessed using the Cochrane ROB 2.0 tool. The identified interventions focused on two approaches to supporting affected others: interventions involving both the problem gambler and affected others, and interventions involving affected others only. As the interventions and outcome measures used were sufficiently similar, a meta-analysis was conducted. The quantitative synthesis revealed that generally, treatment groups were unable to show greater benefits over control groups. The goal for future interventions aimed at affected others of problem gambling should focus primarily on the wellbeing of affected others. The standardisation of outcome measures and data collection time points for better comparison of future research is needed.

**Supplementary Information:**

The online version contains supplementary material available at 10.1007/s10899-023-10220-3.

## Introduction

The world of gambling has been revolutionised by the increase in availability and ease of access to products from any location and at any time, this has introduced new challenges and risks of harm (Gainsbury et al., 2015). Although not all gambling activities are problematic (Meyer et al., 2009), around 2.3% of the world's population engages in problem gambling (Williams et al., 2012). *Problem gambling* is the behaviour that leads to the reduced control over finances and/or time devoted to gambling, which results in adverse effects on the gambler, their family and/or the community (Neal et al., 2005). Its treatment is often multidimensional, and has significantly improved in recent years (Rizeanu, 2018). However, problem gambling continues to be linked with low treatment entry (Braun et al., 2014; Jackson et al., 2014), and substantial rates of treatment drop-out (Melville et al., 2007; Pfund et al., 2021). Moreover, given reported relapse rates, the effectiveness of available interventions in keeping treatment seekers from re-engaging in problem gambling behaviour appears limited (Abbott et al., 2018).

Untreated problem gambling harms protrude onto several affected others (Kourgiantakis et al., 2013; Langham et al., 2016; Riley et al., 2021). Although the prevalence of affected others has been given little attention in the literature, available figures range between 2% (Wenzel et al., 2008) and 19.3% (Salonen et al., 2014) depending on the definition and methodology used by researchers (Salonen et al., 2014; Svensson et al., 2013). This highlights the need for developing effective interventions aimed at supporting this needy population (Dowling, 2020; Heineman, 1994; Orford, 1994; Steinberg, 1993; Tepperman, 1985).

An early literature review by Kalischuk et al. (2006) focusing on problem gambling and its impact on families was the first to include interventions for affected family members of problem gamblers. Findings emphasised the need for interventions to provide families with up-to-date knowledge and a space to share their experiences (Orford, 1994), skills for dealing with financial hardships (Heineman, 1994; Steinberg, 1993) and to focus interventions on the impacts on family members (Heineman, 1994). Kourgiantakis et al. (2013) conducted a systematic review on problem gambling and families. The authors emphasised the importance and benefits of family involvement in the treatment of problem gambling, even if the problem gambler is not in treatment.

Another systematic review aimed to study treatment entry rates for the Community Reinforcement And Family Training (CRAFT) approach across addictions in North America and Europe (Archer et al., 2020), including three studies about problem gambling treatment. More comprehensive interventions had the highest treatment entry rates. The efficacy of these interventions was influenced by training and supervision of therapists and integrated addiction therapy for affected others. Merkouris et al. (2020) aimed to identify available interventions aimed at affected others across addictions and their characteristics, effectiveness, and durability. Of the forty studies, five dealt with interventions aimed at problem gambling. The study found evidence that face-to-face therapist-guided interventions across addictions were associated with more improved treatment outcomes. A scoping review aimed to map family-focused interventions used in the treatment of substance abuse and problem gambling and identify their characteristics and any related gaps (Kourgiantakis et al., 2021). The review concluded that interventions which adopt a clear theoretical framework give the intervention more direction for implementation into practice. Furthermore, interventions need to be more culturally adapted to better meet the needs of the target client group and that there needs to be an increase in training relating to addiction treatment for social workers and professionals working in the field (Kourgiantakis et al., 2018).

A systematic review and meta-analysis aimed to identify the occurrence of Behaviour Change Techniques in psychosocial interventions across the addictions and to determine whether these related to greater effectiveness (Merkouris et al., 2023). The study concluded that further research evaluating the effectiveness of interventions aimed at affected others and using appropriate outcome measures relating to this population is required. Furthermore, future RCTs should include more in-depth information about the delivered interventions. Another study across addictions identified twenty-two studies which did not require the involvement of the addicted individual. Four of these studies related to problem gambling. The statistical synthesis demonstrated mixed findings on the effectiveness of these interventions, highlighting the need for further research in the area. Research on alcoholism remains predominant and its over-representation might introduce potential bias in synthesising studies across addictions (Merkouris et al., 2022).

A scoping review by Dowling et al. (2021) focused on coping strategies, assessment measures and interventions for affected others of problem gambling. Eight studies of interventions aimed at supporting affected others were identified. The review highlighted that access to such services remains low. This might be attributed to the lack of awareness and personal perceptions relating to accessing these services. Furthermore, the questionable effectiveness of treatment over control groups raised uncertainty of whether these interventions are meeting the needs of affected others. The latest systematic review on interventions supporting affected others of problem gambling adopted more lenient inclusion criteria producing a good overview of all available published and grey literature between 2011 and 2021 (Edgren et al., 2021). This review captured various studies which had not been previously included in other reviews, such as low threshold online interventions, qualitative studies, mixed-method studies, and service evaluation studies (Bastardo Gaelzer, 2019; Buchner et al., 2019; Kourgiantakis, 2017; Lee, 2012; Lee, 2015; Orford et al., 2017; Rodda et al., 2017; Shi, 2021). The meta-analysis concluded that none of the interventions showed favourable outcomes over the other except for anxiety and depression. The authors highlighted the importance of appropriate study designs and outcome measurements in future research (Edgren et al., 2021). Nevertheless, results must be interpreted with caution given the amount of data obtained from studies which had not been randomised and unpublished studies increasing the possibility of bias.

## The Importance of this Systematic Review

Problem gambling has been acknowledged as a significant public health concern (Korn et al., 2003; Marshall, 2009). Moreover, a Swedish study by Hofmarcher et al. (2020) noted that the country's societal costs of problem gambling included €38.58 million for the treatment of violence and €196.95 million in the treatment of emotional distress experienced by affected others (Hofmarcher et al., 2020). Despite these financial investments, the efficacy of these interventions has been doubted, which merits further research to inform the development of effective interventions specifically tailored for the needs affected others of problem gambling (Dowling, 2020).

To our knowledge, this is the first systematic review to delineate the difference in efficacy between interventions involving both problem gamblers and affected others and interventions focusing only on affected others aimed at minimising the harm caused to affected others of problem gambling.

## Aims of this Research

The main research question for this systematic review was ‘Are psychosocial interventions effective in minimising harms caused to affected others of problem gambling?’.

The objectives of the present review were twofold:

1. To identify the psychosocial interventions in use to minimise harms caused to affected others of problem gambling.
2. To assess the efficacy of these psychosocial interventions in minimising the harm endured by affected others of problem gambling.

## Methodology

### Advisory Panel

The advisory panel included an affected other, a representative from an online gaming company, representatives from the Responsible Gaming Foundation and service providers. Their real-world expertise helped guide the researchers throughout the course of this research and helped ensure a reduction in bias (Uttley & Montgomery, 2017).

### Research Protocol

A research protocol was written to outline the objectives and methods to be adhered to in the execution of the systematic review. This guaranteed transparency throughout the conduction of the research (Tawfik et al., 2019). Once agreed by the authors and the advisory panel, the review protocol was registered on PROSPERO [CRD42021239138].

### Search Strategy

The search strategy is extremely sensitive and aimed to identify all relevant studies that met the eligibility criteria by adopting a systemic and comprehensive approach. It was developed by reading literature on the research topic, noting key words used in existing journals and by adapting previous search strings used in other reviews dealing with interventions for problem gambling. The detailed search strategy adopted can be found in online resource 1. Databases were selected based on their relevance to the research question. Other sources were searched for ‘grey literature’ including unpublished and ongoing studies. Citation searching was conducted to identify any studies which might have been missed during the electronic search. Contact with identified experts and authors in the field was made to identify unpublished and ongoing studies.

### Inclusion and Exclusion Criteria

We only included RCTs written in English testing psychosocial interventions aimed to minimise harm to affected others of problem gambling. Included participants could be problem gamblers or affected others irrespective of their relationship to the problem gambler. Any psychosocial intervention which as a primary aim or as a secondary by product of the intervention resulted in the minimisation of harms caused to affected others of problem gambling was included. These interventions might have included or excluded the direct involvement of affected others. Interventions which focused solely on the problem gamblers and failed to make any reference to the outcomes of the intervention on affected others of problem gambling were excluded.

### Data Extraction and Process

A data extraction sheet was developed based on the recommendations found in the Cochrane handbook for systematic reviews of interventions (Higgins et al., 2021). The following data were extracted from records included in analyses, study details, country, inclusion criteria, exclusion criteria, total sample size, total number and type of participants, demographics of participants, number, and type of participants in intervention, number and type of participants for comparison, relationship to gambler, intervention content, intervention therapists, comparison content, comparison therapists, follow-up, and study outcomes. The main outcomes assessed for the purpose of this review were depression, anxiety, mental distress, negative emotional consequences, negative behavioural consequences, relationship happiness, relationship assessment, and couple adjustment. Two review authors independently extracted data to be used in effect size estimates including sample sizes, means and standard deviations.

### Risk of Bias

The risk of bias assessment in eligible studies was carried out using the Cochrane risk of bias tool, ROB 2. The tool was used to evaluate five domains: randomisation process; deviation from intended interventions; missing outcome data; measurement of the outcome; and selection of the reported result (Higgins et al., 2021). Table 3 describes the estimated potential risk of bias for all included studies.

### Data Synthesis

Characteristics of each study were assessed according to their Population Intervention Comparison and Outcomes (PICO). Similar studies were grouped together and compared. Overall data were combined where effect sizes were available or could be calculated, and where studies were similar in terms of outcomes measured. Multiple random-effects meta-analysis of outcomes were performed based on standardised mean differences (hedges' g). Meta-analysis of outcomes was conducted on each metric separately.

### Ethics

This study was conducted in line with the ethical standards of the University of Malta.

## Results

### Search Results and the Selection Process

**Fig. 1:**
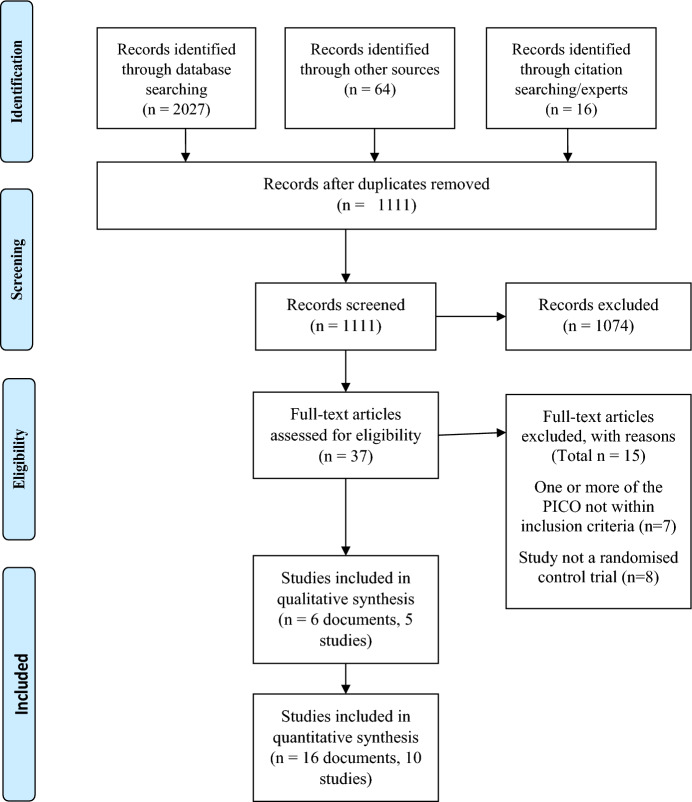
PRISMA statement adapted from https://www.prisma-statement.org/PRISMAStatement/PRISMAStatement

Figure 1 shows the Preferred Reporting Items for Systematic review and Meta-analysis (PRISMA) flowchart, which summarises the search and screening process for this review. A total of 2107 records were identified from different sources outlined in the research protocol. After removing duplicate records, title and abstract screening were carried out for 1111 records, out of which 1074 were excluded. The full-text screening was conducted independently by two review team members on the remaining 37 records; 15 of these records were excluded. A third review team member was involved in helping resolve any conflicts that arose about whether a study met the inclusion criteria set in the review protocol or not. After the collation of reports from the same studies, 5 studies from 6 documents were included in the qualitative synthesis and 10 studies from 16 documents were included in the quantitative synthesis.

### Characteristics of the Included Studies

Of the 15 included studies (N = 983) both qualitative and quantitative studies), the majority (n = 10) evaluated interventions which included both the problem gamblers and affected others, whereas the remaining studies (n = 5) evaluated interventions which included affected others only. Geographically, these studies were predominantly conducted in Canada (n = 10, N = 544), followed by Sweden (n = 4, N = 416) and the USA (n = 1, N = 23). The earliest study dates to 2002, with the most recent studies being published in 2022. All RCTs and any arising articles which fell within the inclusion criteria were included in this review, even if they had already been identified and included in previous reviews. The reason for this was primarily because the aims of this review were different to those of previous reviews; secondly, RCTs of psychosocial interventions aimed at affected others of problem gambling are still in their infancy, and thus including all available studies produced a more holistic picture of all the available evidence to date. The characteristics of the included studies are presented in Tables 1 and 2.

**Table 1:** Characterises of included quantitative studies

Author/year/country	Study design	Recruitment criteria	Number and type of participants	Intervention/control	Therapist training
Makarchuk et al. (2002)/ Makarchuk (2001 – master’s dissertation) Canada	**Design:** 2 parallel RCTs **Outcome data:** Baseline, 3-month follow-up	**Inclusion criteria:** Family member or intimate partner with problem gambling who have minimum contact of 3 days a week. Both gambler and affected other 18 years + and willing to provide follow-up data. Neither the gambler or affected other have received treatment in the last 3 months, and the gambler currently resistant to treatment **Exclusion criteria:** Not meeting eligibility criteria	**Number and type:** 31 affected others **Relationship to gambler:** Spouse (n = 18), boyfriend/girlfriend (n = 4), parent(n = 4), child (n = 4), sibling (n = 1)	**Intervention:** Community Reinforcement and Family Training (CRAFT) manual for Concerned significant others of problem gamblers + control information package **Control:** Treatment as usual information package	**Intervention:** No therapists **Control:** No therapists
Rychtarik and McGillicuddy (2006) USA	**Design:** Delayed treatment RCT **Outcome data:** post-treatment	**Inclusion criteria:** Partner had gambled in the last 3 months, score ≥ 5 on SOGS, no professional or self-treatment for either gambler or the partner in the last 3 months, currently married or living with their partner for at least 1 year, participant score of less than 5 and no evidence of alcohol use disorder or other substance abuse **Exclusion criteria:** Not meeting eligibility criteria	**Number and type:** 23 affected others **Relationship to gambler:** Partners	**Intervention:** 10- week sessions of Coping skills training program (CST) **Control:** 10-week delayed treatment control group (DTC)	**Intervention**: 3 masters-level counsellors received training in administering the manualised treatment **Control:** Same as intervention
Hodgins et al. (2007b)/ Hodgins et al. (2007a)/ Hodgins et al. (2004) Canada	**Design:** 3 parallel RCTs **Outcome data:** Baseline, 3- & 6-month follow-up	**Inclusion criteria:** Both the gamblers and participant 18 years + . In contact with the problem gambler a minimum of 3 days a week. Neither the gambler nor did the participant receive gambling-related treatment in the last 3 months. Gambler was resistant to entering treatment. Gambler was willing and able to read materials written in English. Participant was willing to maintain contact via telephone for 6 months, agreed for telephone calls to be recorded, and provide details of a person the researchers could contact if they couldn't get a hold of the participant **Exclusion criteria:** Not meeting eligibility criteria	**Number and type:** 186 affected others **Relationship to gambler:** spouse (n = 104), child (n = 33), sibling (n = 13), boyfriend/girlfriend (n = 11), parent (n = 11), friend (n = 9), extended family (n = 5)	**Intervention:** (Group 1) Community Reinforcement and Family Training (CRAFT) manual for Concerned significant others of problem gamblers + control information package (Group 2) CRAFT self-help workbook + therapist telephone support (2 scheduled calls) + control information package **Control:** Information package of treatment resources according to participant province	**Intervention:** 3 certified Canadian Problem Gambling Counsellors, had undergrad University degrees and 4 years work experience in the area **Control:** No therapists
Lee and Awosoga (2015) Canada	**Design:** Multi-site pilot RCT **Outcome data:** Baseline, post-treatment (12 weeks), & follow-up (20 weeks)	**Inclusion criteria:** One or both partners had to meet the DSM-IV-TR criteria, gambled in the past 2 months, 18 years + , and in a committed couple relationship **Exclusion criteria:** Suicidal ideation or psychotic symptoms in the last month, recurring intimate partner violence, undergoing other treatment during the study, involved with loan sharks	**Number and type:** 16 gamblers and 14 affected others (both partners in 1 couple were gamblers) **Relationship to gambler:** Partners	**Intervention:** 12- week Congruence Couple therapy (CCT) **Control:** 3 brief check-ins over 12 weeks/ non-specified treatment as usual	**Intervention:** 2 bachelors prepared counsellors and 2 masters prepared counsellors **Control:** check-in calls by research assistant. Potential additional TAU – Not specified
Nayoski and Hodgins (2016)/ Peden (2011- Doctoral Dissertation) Canada	**Design:** 2 parallel RCTs **Outcome data:** Baseline, 3- & 6-month follow-up	**Inclusion criteria:** Both the gamblers and affected others are 18 years + and maintained contact a minimum of 3 days a week. Is a close relative or partner of the gambler. Gambler is resistant to treatment. Gambler meets criteria for problem gambling. Participant reads at a minimum of 6th grade level reading and completes initial face-to-face and follow-up interview via telephone, provide details of a person the researchers could contact if they couldn't get a hold of the participant **Exclusion criteria:** Either the gambler of affected other attended gambling-related treatment in the last 2 months	**Number and type:** 31 affected others **Relationship to gambler:** Spouse (n = 14), common law partner (n = 5), parent (n = 4), child (n = 5), boyfriend/girlfriend (n = 2), separated spouse (n = 1)	**Intervention:** Community Reinforcement and Family Training (CRAFT) manual for Concerned significant others of problem gamblers + 8 to 12—1-h sessions by a therapist to help **Control:** CRAFT self-help workbook completed on a weekly basis	**Intervention:** Master-level therapists (Cognitive-behavioural clinical psychologists) who received 6 h of training on the CRAFT approach **Control:** No therapists
Nilsson et al., (2018) Sweden	**Design:** 2 parallel pilot RCTs **Outcome data:** Baseline, post-treatment, 3- & 6-month follow-up	**Inclusion criteria:** Gambler’s scoring ≥ 8 in PGSI. Affected others no symptoms of gambling and be a partner, family member or a friend knowing each other for a minimum of 3 months. Both living in Sweden, understand and write Swedish and be aged 18 years + **Exclusion criteria:** Severe psychiatric disorders	**Number and type:** 18 gamblers and 18 affected others **Relationship to gambler:** Not specified	**Intervention:** 10 internet-based therapist-guided self-help modules of Behavioural couples therapy (BCT) + weekly telephone and e-mail support from therapist. Affected other received treatment **Control:** 10 internet-based therapist-guided self-help modules of Cognitive behavioural therapy (CBT) + weekly telephone and e-mail support from therapist. Affected other did not receive treatment	**Intervention:** Master-level psychology students and experienced staff from the Swedish National Gambling Helpline. They received training and supervision once every 2 weeks **Control:** Same as intervention
Magnusson et al. (2019) Sweden	**Design:** Wait-list RCT **Outcome data:** Baseline, post-treatment, 3, 6, 12-month follow-up	**Inclusion criteria:** Both the gamblers and affected others are 18 years + , affected others are a parent, child, sibling, friend, or partner of the gamblers. The affected other new the gambler for a minimum of 3 months. Neither the gambler nor affected other received any gambling related treatment in the last 3 months. Gambler is resistant to enter gambling treatment. Affected other can read and answer in Swedish and willing to have telephone contact with a counsellor weekly during the 10 weeks. Gambler’s scoring ≥ 8 in PGSI. Affected others on psychotropic treatment should be stable for at least 3 months **Exclusion criteria:** affected other scoring ≥ 8 in PGSI. Affected other has current psychotic or bipolar disorder	**Number and type:** 100 affected others **Relationship to gambler:** Parent (n = 43), partner (n = 43), other (n = 10)	**Intervention:** 9 modules of Online CRAFT inspired CBT **Control:** Wait-list – same as intervention	**Intervention:** 3 Counsellors instructed on motivational interviewing and 1 master’s level clinical psychologist + weekly supervision **Control:** Same as intervention
Nilsson et al. (2020)/ Nilsson (2019; Doctoral Dissertation) Sweden	**Design:** 2 parallel RCTs **Outcome data:** Baseline, post-treatment, 3, 6, 12-month follow-up	**Inclusion criteria:** Gambler’s scoring ≥ 5 in PGSI. Affected others no symptoms of gambling and be a partner, family member or friend knowing each other for a minimum of 3 months. Both living in Sweden, understand and write Swedish and 18 years + **Exclusion criteria:** Severe psychiatric disorders	**Number and type:**136 gamblers and 136 affected others **Relationship to gambler:** Partner (n = 69), Parent (n = 49) or other (n = 18)	**Intervention:**10 internet-based therapist guided self-help modules of Behavioural couples therapy (BCT) + weekly telephone and e-mail support from therapist. Affected other received treatment **Control:** 10 internet-based therapist-guided self-help modules of Cognitive behavioural therapy (CBT) + weekly telephone and e-mail support from therapist. Affected other did not receive treatment	**Intervention:** 1 licensed psychologist, 3 psychologists in their final year of training, 4 counsellors. They received supervision once every 2 weeks **Control:** Same as intervention
Lee et al. (2022)/ Lee, Ofori Dei & Isik (2022) Canada	**Design:** Non-blinded parallel RCT **Outcome data:** Baseline, post-treatment (5 months) and follow-up at 8-months	**Inclusion criteria:** One or both partners in the couple met the DSM-5 criteria for Alcohol use or Gambling Disorder in the past year. Both were over 18 years of age. Both partners were committed to their relationship **Exclusion criteria:** Either party experienced suicidal thoughts/attempts over the past year, experienced psychotic symptoms in the past month, recurring intimate partner violence, involvement with loan sharks	**Number and type:** 7 couples with one of the partners positive for gambling disorder or both gambling disorder and Alcohol use **Relationship to gambler:** Intimate couples	**Intervention:** Average of 13 systemic congruence couples therapy sessions **Control:** Treatment as usual which included various forms of treatment but excluded any form of couple’s therapy	**Intervention:** 4-day in-person experiential training. CCT delivered by 5 counsellors who had bachelor’s and master’s in social work, addiction counselling and counselling psychology. Counsellors attended weekly group phone conferences for case consultation and submitted ongoing case notes for reviews and comments to ensure adherence **Control:** Not available
Tremblay et al. (2022) Canada	**Design:** Parallel RCT **Outcome data:** Baseline, 4 months post-admission, 10 months post-admission	**Inclusion criteria:** couples living together for a minimum of 6 months, both over 18 years old, one member diagnosed with gambling disorder, the gambler must have gambled in the last 3 months, the gambler shouldn’t have participated in gambling disorder treatment in the last 6 months **Exclusion Criteria:** considerable dependence on alcohol or drugs, both members diagnosed with gambling disorder, history of physical violence, the presence of a psychotic disorder/psychiatric hospitalization, considerable antisocial characteristics in one of the members, presence of suicidal ideation/suicidal attempts, low dyadic commitment in one of the members	**Number and type:** 80 couples **Relationship to gambler:** cohabitating couples	**Intervention:**8–12 sessions of ICT-PG lasting 75 min each **Control:** 15–20 TAU (individual treatment) individual or group sessions lasting 50 min each	**Intervention:** 39 therapists working in addiction centers who received 17 h of ICT-PG training **Control:** Not available

**Table 2:** Characteristics of included qualitative studies

Author/year/country	Study design	Recruitment criteria	Number and type of participants	Intervention/control	Therapist training
Tremblay et al. (2018)/ Tremblay et al. (2015) Canada	Qualitative semi-structured interviews 9 months after admission to treatment	**Inclusion:** Both 18 years + and living together for more than 6 months. One of the members had to be diagnosed with pathological gambling and sought help from any of the centres participating in the study **Exclusion:** Couples with alcohol or drug abuse problems	**Intervention:** 13 couples who received ICT-PG **Control:** 8 couples who received individual treatment	**Intervention:** between 8 to12, 90-min sessions using integrative couple treatment for pathological gambling (ICT-PG) **Control:** Individual treatment for both affected others and problem gamblers	**Intervention:** Not specified **Control:** Not specified
Bastardo Gaelzer (2019- Master’s dissertation) Canada	Secondary analysis of treatment notes from Lee and Awosoga (2015)	**Inclusion:** Treatment notes for the 2 couples with the most favorable treatments outcomes on DAS measures **Exclusion:** None	2 couples	**Intervention:** 12- week Congruence Couple therapy (CCT) **Control:** Not applicable	**Intervention:** 2 bachelors prepared counsellors and 2 masters prepared counsellors **Control:** Not applicable
Nilsson et al. (2021) Sweden	Qualitative semi-structured interviews 2 to 3 years after an RCT (Nilsson et al., 2020)	**Inclusion:** affected other dropped out of treatment **Exclusion:** None	A purposive sample of 4 participants from intervention and 4 participants from control	**Intervention:** Internet-based therapist guided self-help modules of Behavioural couples therapy (BCT) + weekly telephone and e-mail support from therapist. Affected other received treatment **Control:** 10 internet-based therapist-guided self-help modules of Cognitive behavioural therapy (CBT) + weekly telephone and e-mail support from therapist. Affected other did not receive treatment	**Intervention:** 1 licensed psychologist, 3 psychologists in their final year of training, 4 counsellors. They received supervision once every 2 weeks **Control:** Same as intervention
Shi (2021- Master’s dissertation) Canada	Post-treatment interviews with couples from Lee et al., (2022 – unpublished)	**Inclusion:** The couple had completed CCT in the RCT intervention group	2 couples	**Intervention:** Average of 13 systemic congruence couples therapy sessions	**Intervention:** 4-day in-person experiential training. CCT delivered by 5 counsellors who had bachelor’s and master’s in social work, addiction counselling and counselling psychology. Counsellors attended weekly group phone conferences for case consultation and submitted ongoing case notes for reviews and comments to ensure adherence
Lee and Merali (2022) Canada	Thematic qualitative analysis of client case notes arising from an RCT (Lee et al., 2022/ Lee Lee, Ofori Dei & Isik 2022- unpublished manuscript)	**Inclusion:** Case notes of couples included in the CCT intervention group who mentioned any connections relating to employment **Exclusion:** Couples who had no mention of employment related themes in their case notes	21 couples in the CCT intervention group featured employment. These couples included couples with alcohol use disorder, gambling disorder, or both. Only data relating to problem gambling will be utilized for the purpose of this review	**Intervention:** Average of 10 systemic congruence couples therapy sessions	**Intervention:** 4-day in-person experiential training. CCT delivered by 5 counsellors who had bachelor’s and master’s in social work, addiction counselling and counselling psychology. Counsellors attended weekly group phone conferences for case consultation and submitted ongoing case notes for reviews and comments to ensure adherence

### Description of the Psychosocial Interventions in Included Studies

#### Interventions Including both the Problem Gambler and Affected Other

Five RCTs (n = 512) evaluated the efficacy of interventions in the treatment of problem gambling, including both the gambler and the affected other; these interventions included different forms of couple’s therapy (Lee & Awosoga, 2015; Lee et al., 2022; Nilsson et al., 2018, 2020; Tremblay et al., 2022). Nilsson et al., (2018) pilot tested the efficacy of internet-delivered behavioural couples therapy (BCT) against a control group of cognitive behavioural therapy (CBT), both consisting of 10 sessions over 12 weeks. The BCT intervention was inspired by an existing BCT treatment for alcohol and substance use (O'Farrell & Fals-Stewart, 2006). Adapting treatments from other disorders has been a common occurrence in this field of research. Consequently, another couple’s therapy intervention was inspired by the alcohol behaviour couple therapy (ABCT) (McCrady & Epstein, 2009) to develop an intervention for couples impacted by problem gambling (Tremblay et al., 2022).

Nilsson et al., (2020) evaluated the same intervention in a full-scale randomised controlled trial. In both studies, affected others did not receive any form of treatment in the control group. In the pilot study (Nilsson et al., 2018), 18 couples were randomised into either a BCT treatment group or a control group. Post-treatment outcomes for affected others in the BCT intervention group improved from moderate depression and anxiety to no depression and anxiety. At post-treatment, their counterparts in the control group showed no significant improvement. Moreover, they deteriorated in both depression and anxiety at 3-months. At the 6-month follow-up, affected others in the control group returned to earlier levels in both anxiety and depression. In Nilsson et al. (2020), 136 problem gamblers and their affected others, including partners, parents and other individuals, were randomised in the same manner as the pilot study. The only favourable outcomes from the BCT intervention group were a decrease in negative consequences of gambling (ICS) when compared to the control CBT.

Lee and Awosoga (2015) randomised 15 couples into either a Congruence Couples Therapy (CCT) treatment group which consisted of 12-weekly sessions, or the control group, in which couples were advised about self-care plans and contacted by a research assistant once every three weeks. In addition, couples randomised to the control group were allowed to seek counselling while acting as controls if they desired to do so. The post-treatment results showed significant improvements in mental distress (BSI) and system functioning (STIC) in both affected others in the CCT treatment group and the control group. This might indicate that other counselling methods which control couples attended may have been equally successful in improving these outcomes. However, neither group noted a significant improvement in couples’ relationship (DAS) post-treatment or at follow-up. More recently, Lee et al. (2022) conducted a non-blinded RCT using CCT in the treatment of alcohol use and gambling disorders which randomised 46 couples to either CCT or treatment as usual. Only seven couples from the total randomised sample included a partner experiencing either problem gambling only or both problem gambling and alcohol use disorder. A total of four couples were randomised to the treatment group and three couples to the control group, which were included in the final analysis of the study. The available manuscripts for this RCT include collated data of all 46 couples, including couples not experiencing any gambling problems. Thus, no generalisations could be based on this due to the potential differences in impacts experienced between affected others of alcohol users and those of problem gambling. Nevertheless, outcome data for the seven couples impacted by at least problem gambling were obtained through personal communication with the primary author and were included in the quantitative synthesis.

#### Interventions Including the Affected Other only

The second group of five RCTs (n = 371) evaluated interventions including the affected others only. These interventions primarily attempted to equip affected others with a set of coping skills to alleviate the harm experienced because of someone else's problem gambling (Hodgins et al., 2007a; Magnusson et al., 2019; Makarchuk et al., 2002; Nayoski & Hodgins, 2016; Rychtarik & McGillicuddy, 2006). The studies were similar in teaching affected others coping skills through different formats (self-help manual, telephone support, therapist-guided), and problem gamblers were not included in the intervention. Furthermore, neither the problem gambler nor the affected other was in treatment or had received any gambling-related treatment in the three months prior to the study (Hodgins et al., 2007a; Makarchuk et al., 2002; Nayoski & Hodgins, 2016; Rychtarik & McGillicuddy, 2006).

All interventions, except for Rychtarik and McGillicuddy (2006), were available to various affected others as long as they maintained a close relationship with the problem gambler and had minimum contact of three days a week. Three studies used an intervention manual based on a modified version of the CRAFT intervention which was initially developed for affected others of individuals who abused alcohol (Hodgins et al., 2007a; Makarchuk et al., 2002; Nayoski & Hodgins, 2016). Another study delivered 9-online CBT modules for affected others, which were also inspired by the CRAFT approach (Magnusson et al., 2019). The fifth study used a manual based on the stress and coping model. However, limited information about this intervention was available in the published study (Rychtarik & McGillicuddy, 2006).

Two studies had constant guidance by master-level therapists who delivered coping skills training (Peden, 2011; Nayoski & Hodgins, 2016; Rychtarik & McGillicuddy, 2006). Another study scheduled two telephone calls over the 10-week duration of the intervention to help guide affected others (Hodgins et al., 2007a). One study had counsellors who were trained in motivational interviewing, and another study did not have any involvement of any professionals since the intervention was of a self-help format (Makarchuk et al., 2002).

### Risk of Bias

As displayed in Table 3, most studies were classified as having some concerns in their overall risk for bias (80%; n = 8), with the remaining studies being considered as having a high risk of bias (20%; n = 2).

**Table 3:** Risk of Bias of included quantitative studies

Study details	Randomisation	Deviations from the intended interventions	Missing outcome data	Outcome measurement	Selection of reported result	Overall risk of bias
Makarchuk et al. (2002)/ Makarchuk (2001 – master’s dissertation)	Some concerns	High	Low	Low	Some concerns	High
Rychtarik and McGillicuddy (2006)	High	Low	Low	Low	Some concerns	High
Hodgins et al. (2007b)/Hodgins et al.(2007a)/Hodgins et al. (2004)	Some concerns	Low	Low	Low	Some concerns	Some concerns
Lee and Awosoga (2015)	Low	Low	Low	Some concerns	Low	Some concerns
Nayoski and Hodgins (2016)/ Peden (2011- Doctoral Dissertation)	Low	Low	Low	Low	Some concerns	Some concerns
Nilsson et al., (2018)	Low	Low	Low	Some concerns	Low	Some concerns
Magnusson et al. (2019)	Low	Low	Low	Some concerns	Low	Some concerns
Nilsson et al. (2020)/ Nilsson (2019; Doctoral Dissertation)	Low	Low	Low	Some concerns	Low	Some concerns
Lee et al. (2022)/ Lee, Ofori & Isik (2022)	Low	Low	Low	Some concerns	Low	Some concerns
Tremblay et al. (2022)	Low	Low	Low	Some concerns	Low	Some concerns

### Quantitative Synthesis – Efficacy of Psychosocial Interventions in Minimising Harm Caused to Affected Others of Problem Gambling

Of the ten included quantitative studies (n = 883), 9 provided sufficient data for inclusion in the meta-analysis. The study excluded from the quantitative synthesis was the first RCT conducted in the field (Makarchuk et al., 2002). Furthermore, the study by Hodgins et al. (2007a) was a 3-arm RCT. For data synthesis, the data from the workbook group was removed because the other two arms were more similar to those used in Nayoski and Hodgins (2016), which was the primary study with which outcome data was synthesised. Quantitative synthesis was conducted using RevMan version 5.4. A description of the full synthesis can be found in online resource 2.

### Efficacy of Interventions Including both the Problem Gamblers and Affected Others

The quantitative synthesis for efficacy of interventions including the problem gamblers and affected others in minimising affected others' harms was conducted in four outcome domains: depression, anxiety, couple adjustment and mental distress. Outcome measures for depression favoured the intervention group post-intervention (− 0.09) and at the 6-month follow-up (− 0.22), however heterogeneity between the studies was substantial (I^2^ = 65–87%). At the 3-month follow-up results favoured the control group (0.33) with no heterogeneity between studies (I^2^ = 0%). Outcome measures for anxiety favoured control groups. Data synthesis for couple adjustment favoured the control group at post-intervention (0.29, I^2^ = 75%) but favoured the intervention group at the 3-month follow-up (− 0.40, I^2^ = 26%). None of these results were not statistically significant (*p* = 0.06–0.95). For mental distress data synthesis showed a moderate effect size favouring the intervention group at post-intervention (− 0.73) which was statistically significant (*p* = 0.001) with no heterogeneity between studies (I^2^ = 0%).

### Efficacy of Interventions Including the Affected Other Alone

The quantitative synthesis for efficacy of interventions including affected others only in minimising affected others' harms was conducted in seven outcome domains: depression, anxiety, negative emotional consequences, negative behavioural consequences, mental distress, relationship happiness and relationship assessment. Outcome data favoured intervention groups in two domains, namely depression and anxiety. For depression, a small effect size (− 0.49) favouring the intervention was noted post-intervention. The heterogeneity between the studies was moderate (I^2^ = 33%). Overall effect showed no statistical significance (*p* = 0.09). Both studies were wait-list RCTs; thus, no further synthesis could be made to study this finding as no outcome data for the control group was available beyond this time point. For anxiety, data synthesis revealed a medium effect size (− 0.59) post-intervention favouring the intervention group. However, this was not statistically significant (*p* = 0.23). Furthermore, heterogeneity between the studies was substantial (I^2^ = 72%).

### Comparison of Efficacy Between Interventions Including both the Problem Gambler and Affected Others, and Interventions Including Affected Others Alone

Due to the inconsistency in outcome measures used and data-collection time points, a comparison between the efficacy of interventions including both problem gamblers and affected others as well as interventions including affected others alone in minimising affected others' harms could only be made for two outcome domains, namely anxiety and depression. Moreover, this inconsistency made a comparison of these outcome domains only possible post-intervention. For anxiety, interventions including affected others only, showed better results at post-intervention (− 0.59) compared to interventions including both problem gamblers and affected others which showed no significant difference between intervention and control groups. For depression, interventions including affected others only showed better results at post-intervention (− 0.49), favouring the intervention group when compared to interventions including both problem gamblers and affected others (− 0.09).

## Qualitative Synthesis

### Included Studies and Analytical Themes

The results section of five studies (Bastardo Gaelzer, 2019; Lee & Merali, 2022; Nilsson et al., 2021; Shi, 2021; Tremblay et al., 2018) which met the inclusion criteria were analysed for qualitative data pertaining to the treatment process of affected others of problem gambling. All five studies dealt with interventions involving both the problem gamblers and affected others namely through CCT, ICT-PG and BCT. Subsequently, five analytical themes were derived: treatment needs, treatment benefits, treatment facilitators, treatment barriers and implications for future treatment.

### Treatment Needs

The importance of treatment in meeting the needs of affected others was expressed in several instances. The need for better understanding of the problem gambler's experiences and vice versa was common, particularly concerning the recovery process. It was also crucial for affected others to understand the psychology behind the gambler’s addiction, this increased the possibility of mutual support (Bastardo Gaelzer, 2019; Tremblay et al., 2018). The need for practical communication skills was also noted. Participants expressed that due to ineffective communication, they could not speak openly about the difficulties arising from problem gambling (Bastardo Gaelzer, 2019; Tremblay et al., 2018). Affected others also needed a space to talk and share their experiences and issues (Tremblay et al., 2018).

### Treatment Benefits

Understanding the gamblers' triggers and urges to gamble led affected others to be less judgemental about the gamblers’ addiction which improved their relationship, and allowed them to better assist in dealing with, and preventing relapse (Bastardo Gaelzer, 2019; Tremblay et al., 2018). Interventions provided a space for the couple to speak about their feelings and emotions, which improved the understanding of each other’s experiences. Moreover, the presence of a neutral person during couples therapy aided open and constructive communication. Discussions started during therapy sessions often continued after the sessions improving communication between the affected other and the problem gambler outside of the therapeutic environment (Bastardo Gaelzer, 2019; Tremblay et al., 2018). Through couples therapy, participants became more aware of the need to dedicate more time to their families and to achieve better work-life balance (Lee & Merali, 2022). Rekindling simple couple activities helped enhance their relationship with the problem gambler (Bastardo Gaelzer, 2019; Tremblay et al., 2018). Another benefit associated with couples therapy was that it enhanced the problem gamblers' commitment to attending regular treatment and made it possible for affected others to receive the support they needed (Tremblay et al., 2018).

### Treatment Facilitators

Building a therapeutic alliance with clients ensured that the therapist gained their trust and understood their expectations which allowed the therapist to better plan future sessions. Exploring the client's needs and wishes also created a positive-therapeutic environment, as opposed to focusing on the negative notions that are typically associated with needing to attend therapy (Bastardo Gaelzer, 2019).

### Treatment Barriers

Interventions were primarily generic and not tailored to the specific needs of the individual. Consequently, upon achieving their treatment goals, participants felt they no longer needed to continue participating in the intervention (Nilsson et al., 2021). Another barrier of couples therapy is that it limits self-expression. As the focus is often to enhance the relationship between the affected other and the problem gambler, there is no space for targeting individual issues. Furthermore, because the couples had to attend therapy sessions jointly, this caused practical issues such as conflicting schedules which increased the possibility of treatment dropout (Shi, 2021).

### Implications for Future Treatment

Although most couples were satisfied with the intervention they received, some participants expressed that a combination of both individual interventions and couples therapy would be more beneficial. Participants suggested that initially they should receive separate treatment, moving onto couples therapy later as this would allow them to speak more openly about their experiences without negatively impacting the problem gambler. Furthermore, individual interventions might better equip them to understand the psychology of problem gambling, with couples therapy focusing more on the problems in their relationship. Other participants expressed that attending treatment independently would allow them to progress at their own pace (Tremblay et al., 2018).

## Discussion

The research question this systematic review sought to answer was “Are psychosocial interventions effective in minimising harms caused to affected others of problem gambling?”. This was primarily achieved by identifying the psychosocial interventions to minimise the harm caused to affected others of problem gambling and then assessing and comparing their outcomes.

### The Psychosocial Interventions Supporting Affected Others of Problem Gambling

Despite the various impacts incurred by affected others of problem gambling (Langham et al., 2016) and the knowledge that their prevalence is much higher than that of problem gamblers (Salonen et al., 2014; Wenzel et al., 2008), available psychosocial interventions aimed at supporting affected others of problem gambling are limited. Consequently, a mere fifteen studies from ten RCTs met the inclusion criteria of this review which involved 883 participants (Archer et al., 2020; Dowling et al., 2021; Edgren et al., 2021; Kalischuk et al., 2006; Kourgiantakis et al., 2021; Kourgiantakis et al., 2013; Merkouris, Downling et al., 2020; Merkouris et al., 2022; Merkouris et al., 2023).

The identified psychosocial interventions took two main approaches: those involving the problem gamblers *and* affected others (Lee & Awosoga, 2015; Lee et al., 2022; Nilsson et al., 2018, 2020; Tremblay et al., 2022) and those involving *affected others alone* (Hodgins et al., 2007a; Magnusson et al., 2019; Makarchuk et al., 2002; Nayoski & Hodgins, 2016; Rychtarik & McGillicuddy, 2006). These interventions were delivered through different modalities, including self-help, therapist-guided, face-to-face, and remotely. Offering diverse treatment delivery options might increase treatment-seeking behaviour since it has been noticed that affected others have different preferences of treatment delivery when seeking support (Buchner et al., 2019; Dowling et al., 2014).

The qualitative analysis suggested that affected others have mixed opinions about both treatment options and suggested future interventions to offer combined interventions (Tremblay et al., 2018). However, individual interventions should be offered first as this allows affected others and problem gamblers to systematically work on their own issues before tackling other issues together. Although other formal (Orford et al., 2017) and low-intensity interventions (Buchner et al., 2019) are available, their efficacy has not been evaluated in RCTs. Low-intensity interventions might attract affected individuals who do not wish to seek more formal and intensive interventions (Buchner et al., 2019; Dowling et al., 2014). Thus, the efficacy of these interventions is also crucial for developing future treatment options.

### The Efficacy of Psychosocial Interventions in Minimising Harm Caused to Affected Others

This systematic review was the first to differentiate between the efficacy of psychosocial interventions involving the problem gamblers and affected others and those involving affected others only. This review concluded that generally, intervention groups were incapable of proving more significant benefits than control groups. Nevertheless, a significant result favouring the intervention group at post-intervention for mental distress was noted from the synthesised quantitative data of two studies involving both the problem gamblers and affected others (Lee & Awosoga, 2015; Tremblay et al., 2022). Quantitative data for mental distress at post-intervention was not available for synthesises for interventions involving affected others alone. In addition to allowing for the comparison of results between these two interventions, better streamlining in reporting outcome measures assessing the efficacy of these interventions would have permitted the inclusion of results from other RCTs which fell within the inclusion criteria. This would have given a better indication of the significance of this result, and its change over time.

A previous review which collated data from studies involving both types of interventions together concluded that they only showed minimal superiority over control groups in outcome domains relating to anxiety and depression (Edgren et al., 2021). The present review was able to deduce that interventions including affected others alone showed slightly better results for anxiety and depression over interventions including both affected others and problem gamblers. A reason for this might be that couples therapy might limit self-expression, and there is no space for affected others to deal with their own issues (Bastardo Gaelzer, 2019). Nevertheless, none of these results were statistically significant, meriting further research to determine the validity of these results.

There may be various other contributory factors to the lack of efficacy of psychosocial interventions in meeting the needs of affected others. Primarily, affected others have multiple treatment needs (Langham et al., 2016), which might be challenging to address concurrently. This is aggravated by the knowledge that affected others often seek professional support when the gambler's addiction has become severe (Järvinen-Tassopoulos, 2020). Thus, the gravity of the repercussions transferred to affected others might be parallel to this. More severe consequences might require more time and multiple interventions to be adequately minimised. One of the most common motivators for support-seeking by affected others is worsened financial impacts (Järvinen-Tassopoulos, 2020) which also results in negative relationship impacts (Langham et al., 2016). Consequently, participants would have liked more financial guidance to be offered from these interventions (Klevan et al., 2019). In view of this, involving financial advisors throughout the development and assessment of future psychosocial interventions aimed at minimising harm caused to affected others of problem gambling might be beneficial in better meeting these needs. Furthermore, the content of the available interventions is often manualised, and thus, it is not tailored to the specific needs of individual clients. Generic interventions might cause treatment-seeking individuals to drop out of treatment once their needs are met (Nilsson et al., 2021). Thus, a client-centred approach should be adopted to meet the specific needs of each individual and make better use of available resources.

The limited number of RCTs evaluating interventions aimed at minimising the harm caused to affected others of problem gambling, subsequently limits the development of evidence-based interventions in the field. Moreover, some of these studies are limited by small sample sizes. Additionally, despite the advancements in the development of outcome measures created explicitly for measuring harm experienced by affected others of problem gambling, such as PG-SOIS (Dowling et al., 2014) and PG-FIM (Dowling & Jackson, 2016), none of the included RCTs used these outcome measure tools. Furthermore, despite the knowledge of prevalence and severity of financial harm experienced by affected others (Li et al., 2016), measurement of this impact is unclear in these studies. This might indicate that previous RCTs did not adequately capture treatment outcomes relating to affected others of problem gambling by using generic psychological outcome measures. Studies showed significant inconsistency in the types of outcome measures used and the time points at which outcome data were collected. Consequently, this limited the meta-analyses in cumulatively assessing the efficacy of these interventions.

Finally, despite the emphasis on the importance of sufficient training delivered to professionals about intervention content and the multifaceted needs of affected others of problem gambling (Campos-Melady et al., 2017; Merkouris et al., 2020), few studies offered training prior to the conduction of the study. Furthermore, studies offering preliminary training did not mention specific content relating to problem gambling.

### Strengths and Limitations of this Review

The major strength of this review lies in the fact that it is the first systematic review and meta-analysis to synthesise all RCTs written in English pertaining to psychosocial interventions aimed at minimising the harm caused to affected others of problem gambling to date. The inclusion of qualitative data from RCTs added value to the review as it better represented what affected others look for when seeking support to deal with repercussions experienced due to someone else's gambling addiction. Furthermore, the randomisation of participants in the included studies, limited bias, even though the review included unpublished studies and student dissertations. Using Cochrane's latest risk of bias tool ROB 2.0 ensured transparency of any potential bias arising from studies included in the quantitative synthesis. This review was also the first to compare the efficacy of psychosocial interventions involving both the problem gamblers and affected others and those including affected others only.

Nevertheless, due to the lack of measurement of affected other outcomes across RCTs, this comparison was limited to depression and anxiety outcomes only. Another potential limitation of this review is that due to limiting searches to studies written in English, RCTs published in other languages which might have otherwise met the inclusion criteria were omitted. This might have compounded the already limited number of available studies that met the inclusion criteria.

### Directions for Future Research

A list of core outcomes that should be measured by studies evaluating psychosocial interventions aimed at minimising the harm caused to affected others by problem gambling would help the field. This will ensure that findings from future research will assess the efficacy of these interventions in minimising the diverse impacts experienced by affected others of problem gambling more rigorously and allow for better generalisation of results arising from future meta-analyses. Additionally, standardisation of data-collection time-points for outcome measures across studies will further enhance the results of future meta-analyses. Future RCTs should also consider engaging a Delphi panel throughout the research process so that research aims, and interventions are better aligned with the needs of affected individuals. Professionals providing these interventions should be offered thorough training prior to delivering the treatment to ensure that service users reap the utmost benefits from receiving the intervention. Future interventions should be tailored to each service user so that the needs of each individual are better met. Lastly, current, and correct information about problem gambling and the available interventions for affected others should be disseminated to the broader body of professionals who might encounter these individuals so that timely referrals are made.

## Conclusion

This systematic review and meta-analysis identified the psychosocial interventions that aimed to minimise the harm caused to affected others by problem gambling and their efficacy as evaluated in several RCTs. The two main types of identified interventions included the problem gambler and the affected other, and those included affected others on their own. Generally, from the findings of this review, it is evident that none of these two groups of interventions effectively minimised the multi-faceted impacts that this population experiences because of someone else's problem gambling more than the control groups. However, results favouring intervention groups over control groups for anxiety and depression post-intervention were observed in interventions involving affected others only. Standardisation of core outcome measures and data-collection time-points is needed to give a better indication of the efficacy of these interventions.

### Supplementary Information

Below is the link to the electronic supplementary material.

Supplementary file1 (PDF 140 KB)

Supplementary file2 (PDF 89 KB)
