# Supplementary material for: The Efficacy of Psychosocial Interventions in Minimising the Harm Caused to Affected Others of Problem Gambling: A Systematic Review and Meta-Analysis
Source: J Gambl Stud. 2023 Jun 9;39(4):1927–58. doi: 10.1007/s10899-023-10220-3 (PMC10627969; doi:10.1007/s10899-023-10220-3)
Supplement: Supplementary file 1 — Supplementary file1 (PDF 140 KB) [file 10899_2023_10220_MOESM1_ESM.pdf]

Online Resource 1 for the efficacy of psychosocial interventions in minimising the harm caused to affected others of problem gambling: A systematic review and meta-analysis: Detailed account of the search strategy

The search strategy

The search strategy aimed to identify all the relevant studies that met the eligibility criteria set in the review protocol adopting a systematic and comprehensive approach. The search string was developed by noting keywords used in the relevant literature and by adapting search strings used in previous reviews dealing with interventions for problem gambling. The search strategy sought to identify relevant studies from sources including bibliographic databases, search engines, trial registers, citation searching and contacting experts. Assistance from a librarian at the University of Malta was employed to ensure that the developed search string could be utilised in the selected databases, and adaptations were made as necessary.

Database search

An extensive search was conducted to limit potential reporting bias and to maximise sensitivity. The bibliographic databases searched were planned at the protocol development stage. Databases were selected based on their relevance to the research question. The databases searched were CENTRAL, MEDLINE, Social Science Database, CINHAL Complete, Academic Search Ultimate and PsycINFO.

Searching other sources

For the search strategy to be as extensive as possible, other sources were searched for 'grey literature', including unpublished and ongoing studies, which further decreased the potential of publication bias. The sources searched were EBSCO Open Dissertations, Google Scholar, Google search engine, ClinicalTrials.gov and WHO International Clinical Trials Registry Platform (ICTRP).

Citation searching and contacts with experts

Citation searching was conducted to identify any studies which might have been missed during the electronic search. Previous reviews on the same topic and reference lists of included studies were hand searched for potential included studies.

An email was sent to 16 identified experts and authors in the field asking for unpublished data that might fall within the study's inclusion criteria. We received a reply from 7 of these experts.

L-Università ta' Malta

Matthew Vassallo <matthew.vassallo@um.edu.mt>

The efficacy of psychosocial interventions in minimising harm caused to affected others of problem gambling

Matthew Vassallo <matthew.vassallo@um.edu.mt> 23 April 2022 at 15:21
Bcc: nicki.dowling@deakin.edu.au, Joel.Tremblay@uqtr.ca, mark.griffiths@ntu.ac.uk, stephanie.merkouris@deakin.edu.au, simone.rodda1@deakin.edu.au, toula.kourgiantakis@utoronto.ca, ub@handlungs-spielraum.de, sabine.haertl@bas-muenchen.de, kristoffer.magnusson@ki.se, j.f.orford@bham.ac.uk, dan.lubman@monash.edu, rychtari@ria.buffalo.edu, a.g.copello@bham.ac.uk, sjimenez@bellvitgehospital.cat

Dear colleague,

I trust this email finds you well. My team and I are currently conducting a systematic review on the **efficacy of psychosocial interventions in minimising harm caused to affected others of problem gambling**. You have been identified as an expert in the field of gambling/addiction. We are looking for unpublished or ongoing research relating to the aforementioned topic. Although our main interest is in randomized controlled trials, any other information would also be greatly appreciated.

Should you wish more information about our review, its protocol can be accessed at https://www.crd.york.ac.uk/prospero/display_record.php?RecordID=239138

My team and I are appreciative of your time and support in assisting us with our research.

Thank you in advance.

Kind regards,
--

L-Università ta' Malta

Matthew Vassallo | Research Support Officer II
B.Sc. (Hons)(Melit.), M. Family St.(Melit.)

Faculty for Social Wellbeing
(He/his)

Database searches

Social science database 25.4.2022

#	Searches	Results
**1**	("affected others" OR spouse* OR "concerned significant others" OR CSO OR famil* OR "loved ones" OR child* OR friend* OR partner* OR parent*)	**1,133,103**
**2**	("problem gambling" OR "pathologic gambling" OR "gambling harms" OR "gambling disorder" OR "gambling addiction")	**772**
**3**	(intervention* OR therap* OR treat* OR counsel* OR ("psychosocial intervention" OR "psychosocial interventions")	**862,790**
**4**	mesh.Exact("Gambling")	**130**
**5**	("affected others" OR spouse* OR "concerned significant others" OR CSO OR famil* OR "loved ones" OR child* OR friend* OR partner* OR parent*) AND ("problem gambling" OR "pathologic gambling" OR "gambling harms" OR "gambling disorder" OR "gambling addiction") AND (intervention* OR therap* OR treat* OR counsel* OR ("psychosocial intervention" OR "psychosocial interventions")	**583**
**6**	(affected others OR spouse* OR concerned significant others OR famil* OR loved ones OR child* OR friend* OR partner* OR parent*) AND (problem gambling OR pathologic gambling OR gambling harms OR gambling disorder OR gambling addiction) AND (intervention* OR therap* OR treat* OR counsel* OR psychosocial intervention*) AND mesh.Exact("Gambling")	**82**

CINHAL complete (EBSCO host) 25.4.2022

#	Searches	Results
**1**	("affected others" OR spouse* OR "concerned significant others" OR CSO OR famil* OR "loved ones" OR child* OR friend* OR partner* OR parent*)	**1,344,819**
**2**	("problem gambling" OR "pathologic gambling" OR "gambling harms" OR "gambling disorder" OR "gambling addiction")	1,343
**3**	(intervention* OR therap* OR treat* OR counsel* OR ("psychosocial intervention" OR "psychosocial interventions")	2,792,317
**4**	("affected others" OR spouse* OR "concerned significant others" OR CSO OR famil* OR "loved ones" OR child* OR friend* OR partner* OR parent*) AND ("problem gambling" OR "pathologic gambling" OR "gambling harms" OR	137

	"gambling disorder" OR "gambling addiction") AND (intervention* OR therap* OR treat* OR counsel* OR ("psychosocial intervention" OR "psychosocial interventions")	

Academic search ultimate 25.4.2022

#	Searches	Results
**1**	("affected others" OR spouse* OR "concerned significant others" OR CSO OR famil* OR "loved ones" OR child* OR friend* OR partner* OR parent*)	**4,253,660**
**2**	("problem gambling" OR "pathologic gambling" OR "gambling harms" OR "gambling disorder" OR "gambling addiction")	**4,256**
**3**	(intervention* OR therap* OR treat* OR counsel* OR ("psychosocial intervention" OR "psychosocial interventions")	**6,072,774**
**4**	("affected others" OR spouse* OR "concerned significant others" OR CSO OR famil* OR "loved ones" OR child* OR friend* OR partner* OR parent*) AND ("problem gambling" OR "pathologic gambling" OR "gambling harms" OR "gambling disorder" OR "gambling addiction") AND (intervention* OR therap* OR treat* OR counsel* OR ("psychosocial intervention" OR "psychosocial interventions")	**587**

Medline complete 25.4.2020

#	Searches	Results
**1**	("affected others" OR spouse* OR "concerned significant others" OR CSO OR famil* OR "loved ones" OR child* OR friend* OR partner* OR parent*)	**4,888,703**
**2**	("problem gambling" OR "pathologic gambling" OR "gambling harms" OR "gambling disorder" OR "gambling addiction")	**3,688,439**
**3**	(intervention* OR therap* OR treat* OR counsel* OR ("psychosocial intervention" OR "psychosocial interventions")	**10,963,566**
**4**	("affected others" OR spouse* OR "concerned significant others" OR CSO OR famil* OR "loved ones" OR child* OR friend* OR partner* OR parent*) AND ("problem gambling" OR "pathologic gambling" OR "gambling harms" OR "gambling disorder" OR "gambling addiction") AND (intervention* OR therap* OR treat* OR counsel* OR ("psychosocial intervention" OR "psychosocial interventions")	**462**

APA psychinfo 25.4.2022

#	Searches	Results
**1**	("affected others" OR spouse* OR "concerned significant others" OR CSO OR famil* OR "loved ones" OR child* OR friend* OR partner* OR parent*)	**1,452,921**
**2**	("problem gambling" OR "pathologic gambling" OR "gambling harms" OR "gambling disorder" OR "gambling addiction")	**6,153**
**3**	(intervention* OR therap* OR treat* OR counsel* OR ("psychosocial intervention" OR "psychosocial interventions")	**1,686,730**
**4**	("affected others" OR spouse* OR "concerned significant others" OR CSO OR famil* OR "loved ones" OR child* OR friend* OR partner* OR parent*) AND ("problem gambling" OR "pathologic gambling" OR "gambling harms" OR "gambling disorder" OR "gambling addiction") AND (intervention* OR therap* OR treat* OR counsel* OR ("psychosocial intervention" OR "psychosocial interventions")	**744**

Additional Sources

EBSCO open dissertations 25.4.2022

#	Searches	Results
**1**	("affected others" OR spouse* OR "concerned significant others" OR CSO OR famil* OR "loved ones" OR child* OR friend* OR partner* OR parent*)	**156,713**
**2**	("problem gambling" OR "pathologic gambling" OR "gambling harms" OR "gambling disorder" OR "gambling addiction")	**80**
**3**	(intervention* OR therap* OR treat* OR counsel* OR ("psychosocial intervention" OR "psychosocial interventions")	**183,925**
**4**	("affected others" OR spouse* OR "concerned significant others" OR CSO OR famil* OR "loved ones" OR child* OR friend* OR partner* OR parent*) AND ("problem gambling" OR "pathologic gambling" OR "gambling harms" OR "gambling disorder" OR "gambling addiction") AND (intervention* OR therap* OR treat* OR counsel* OR ("psychosocial intervention" OR "psychosocial interventions")	**24**

Google scholar 25.4.2022

First 2 pages of the search

Search

intervention problem gambling concerned significant others affected others treatment therapy support

20 results = **8 relevant items**

Google search engine 25.4.2022

First 2 pages of the search

Search

intervention problem gambling concerned significant others affected others treatment therapy support

20 results = **7 relevant items**

Citation Search and Contact with experts

2 documents were identified through citation searching and 16 documents were identified through contact with experts.

In addition to the 16 documents identified through communication with identified experts, it was also revealed that another RCT study focusing on psychosocial interventions aimed at minimising harm caused to affected others of problem gambling is currently being carried out by Stephanie Merkouris.
