# Supplementary material for: The Efficacy of Psychosocial Interventions in Minimising the Harm Caused to Affected Others of Problem Gambling: A Systematic Review and Meta-Analysis
Source: J Gambl Stud. 2023 Jun 9;39(4):1927–58. doi: 10.1007/s10899-023-10220-3 (PMC10627969; doi:10.1007/s10899-023-10220-3)
Supplement: Supplementary file 2 — Supplementary file2 (PDF 89 KB) [file 10899_2023_10220_MOESM2_ESM.pdf]

Online Resource 2 for the efficacy of psychosocial interventions in minimising the harm caused to affected others of problem gambling: A systematic review and meta-analysis: Detailed account of the quantitative synthesis

Quantitative synthesis – Efficacy of psychosocial interventions in minimising harm caused to affected others of problem gambling

Of the ten included quantitative studies, nine provided sufficient data for inclusion in the meta-analysis. The authors of the tenth study were contacted via email and asked to provide the missing data so the study could be quantitatively synthesised. However, the necessary data for inclusion in the quantitative synthesis had not been collected during their study and, thus, had to be excluded. The study excluded from the quantitative synthesis was the first RCT conducted in the field; although the study was part of a master's dissertation, its findings were also published in a peer-reviewed article (Makarchuk, Hodgins, & Peden, 2002). Furthermore, the study by Hodgins et al. (2007) was a 3-arm RCT. For data synthesis, the data from the workbook group was removed because the other two arms were more similar to those used in Nayoski and Hodgins (2016), which was the primary study with which outcome data was synthesised.

Efficacy of interventions including both the problem gamblers *and* affected others

The efficacy of interventions, including the problem gamblers and affected others in minimising affected others' harms, are presented according to each outcome domain (depression, anxiety, mental distress, and couple adjustment).

Depression

Three studies (Lee et al., 2022; Nilsson et al., 2020; Nilsson et al., 2018) measured depression using PHQ-9 and one study (Tremblay et al., 2022) measured depression using CES-D as the data-collection instrument. Data synthesis showed small effect sizes favouring the intervention group post-intervention (-0.09), and 6-month follow-up (-0.22). Tremblay et al. (2022) was not included in the 3-month follow-up synthesis since no data was collected at this timepoint. Data synthesis at the 3-month follow-up showed a small effect size favouring the control group (0.33). None of these results were statistically significant ($p = 0.06$-0.76).

Anxiety

GAD-7 was used by Nilsson and colleagues (2018, 2020) to measure anxiety. Effect sizes at post-intervention and the 3-month follow-up were non-significant (0.03-0.12). However, in the 6-month follow-up, data showed a small effect size (0.22), favouring the control group. Heterogeneity between the studies substantial at the 3-month follow-up ($I^2 = 60\%$) and moderate at the 6-month follow-up ($I^2 = 36\%$).

Couple adjustment

Three studies measured Couple adjustment using DAS (Lee & Awosoga, 2015; Lee et al., 2022; Tremblay et al., 2022). Data synthesis showed a small effect size favouring the control group at post-intervention (0.29). Tremblay et al. (2022) was not included in the 3-month follow-up synthesis since no data was collected at this timepoint. Data synthesis at the 3-month follow-up showed a small effect size favouring the intervention group (-0.40). None of these results were statistically significant ($p = 0.48$-0.62).

Mental distress

Two studies measured mental distress (Lee & Awosoga, 2015: Tremblay et al., 2022) using BSI and IDPESQ as outcome measures respectively. Data synthesis showed a moderate effect size favouring the intervention group at post-intervention (-0.73) which was statistically significant ($p = 0.001$).

Efficacy of interventions including the affected others alone

The efficacy of interventions including affected others only in minimising affected others' harms are presented according to each outcome domain (depression, anxiety, negative emotional consequences, negative behavioural consequences, mental distress, relationship happiness and relationship assessment).

Depression

Two studies measured depression post-intervention, one using BDI-II (Rychtarik & McGillicuddy, 2006) and one using PHQ (Magnusson et al., 2019). A small effect size (-0.49) favouring the intervention was noted post-intervention. The heterogeneity between studies was moderate ($I^2 = 33\%$). Overall effect showed no statistical significance ($p = 0.09$). Both studies were wait-list RCTs; thus, no further synthesis could be made to analyse this finding as no outcome data for the control group was available beyond this time point.

Anxiety

Two studies measured anxiety post-intervention, one using BAI (Rychtarik & McGillicuddy, 2006) and one using GAD-7 (Magnusson et al., 2019). Data synthesis revealed a medium effect size (-0.59) post-intervention; however, this was not statistically significant ($p = 0.23$). Furthermore, heterogeneity between studies was substantial ($I^2 = 72\%$).

Negative emotional consequences

Two studies using ICS measured negative emotional consequences arising from problem gambling (Hodgins et al., 2007; Nayoski & Hodgins, 2016). At the 3-month follow-up, a small effect size (0.43) favouring the control group was noted; this result showed some statistical significance ($p = 0.03$). At the 6-month follow-up, a small effect size (-0.28) favoured the intervention group; however, this was not statistically significant ($p = 0.71$). Moreover, heterogeneity between the studies was substantial for the latter ($I^2 = 89\%$).

Negative behavioural consequences

Two studies used ICS to measure negative behavioural consequences (Hodgins et al., 2007; Nayoski & Hodgins, 2016). Data synthesis at the 3-month follow-up showed a small effect size (0.39) which favoured the control. However, this was not statistically significant ($p = 0.36$). At the 6-month follow-up, there was a minimal difference between the intervention and control group. Furthermore, at this time point, the heterogeneity between studies was substantial ($I^2 = 68\ \% - 79\%$).

Mental distress

Two studies measured mental distress using BSI (Hodgins et al., 2007; Nayoski & Hodgins, 2016). A medium effect size (0.55) at the 3-month follow-up and small effect size (0.26) at the 6-month follow-up both favoured the control group. However, these findings were both statistically non-significant. Furthermore, substantial heterogeneity between studies was noted at the 3-month follow-up ($I^2 = 87\%$).

Relationship happiness

Two studies used RHS to measure relationship happiness (Hodgins et al., 2007; Nayoski & Hodgins, 2016). No significant difference between intervention and control groups was noted at the 3-month follow-up. A medium effect size (0.56) favouring the control group was seen at the 6-month follow-up. However, once again the heterogeneity between the findings of these studies was substantial ($I^2 = 84\%$).

Relationship assessment

Two studies used RAS to measure relationship assessment (Hodgins et al., 2007; Nayoski & Hodgins, 2016). No significant difference between intervention and control groups was noted at the 3-month follow-up. A small effect size (0.43) favouring the control group was observed at the 6-month follow-up. However, heterogeneity between studies was substantial ($I^2 = 89\%$).

Comparison of efficacy between interventions including both the problem gambler and affected others, and interventions including affected others alone

Due to the inconsistency in outcome measures used and data-collection time points, a comparison between the efficacy of interventions including both problem gamblers and affected others and interventions including affected others alone in minimising affected others' harms could only be made for two outcome domains: anxiety and depression. Moreover, this inconsistency made a comparison of these outcome domains only possible post-intervention.

Anxiety

Two studies, including the problem gamblers and affected others, measured outcome data for anxiety at post-intervention using GAD-7 (Nilsson et al., 2020; Nilsson et al., 2018). Two studies including affected others only in treatment measured outcome data for anxiety at post-intervention using BAI (Rychtarik & McGillicuddy, 2006) and GAD-7 (Magnusson et al., 2019). Interventions including affected others only showed better results at post-intervention (-0.59) compared to interventions including both problem gamblers and affected others which showed no significant difference between intervention and control groups.

Depression

Four studies including the problem gamblers and affected others measured outcome data for depression post-intervention, three using PHQ (Lee et al., 2022; Nilsson et al., 2020; Nilsson et al., 2018) and one using IDPESQ (Tremblay et al., 2022). Two studies including affected others only measured this outcome data using BDI-II (Rychtarik & McGillicuddy, 2006) and PHQ (Magnusson et al, 2019). Data synthesis revealed that both interventions showed a small effect size favouring the intervention group. However, interventions including affected others alone showed slightly better results favouring the intervention group (-0.49), when compared to interventions including both problem gamblers and affected others (-0.09). Nevertheless, neither result was statistically significant ($p = 0.09$-0.76).

List of acronyms for outcome measures

**Acronym**	**Outcome measure**
BDI-II	Beck Depression Inventory-II
PHQ	Patient Health Questionnaire
BAI	Beck Anxiety Inventory
GAD-7	Generalised Anxiety Disorder Assessment
ICS	Inventory of Consequences
BSI	Brief Symptom Inventory
RHS	Relationship Happiness Scale
RAS	Relationship Assessment Scale
DAS	Dyadic Adjustment Scale
CES-D	Center for Epidemiologic Studies Depression Scale
IDPESQ	Psychological Distress Index
